# Outcomes of Esophageal Varices in Adults With Fontan Palliation and Liver Cirrhosis

**DOI:** 10.1016/j.cjcpc.2024.01.002

**Published:** 2024-02-03

**Authors:** Marwan H. Ahmed, William R. Miranda, Patrick S. Kamath, Moira H. Sugrue, C. Charles Jain, Maan Jokhadar, Luke J. Burchill, Heidi M. Connolly, Alexander C. Egbe

**Affiliations:** a Department of Cardiovascular Medicine, Mayo Clinic, Rochester, Minnesota, USA; b Division of Gastroenterology and Hepatology, Mayo Clinic, Rochester, Minnesota, USA

## Abstract

**Background:**

The purpose of this study was to define the risk and outcomes of esophageal varices in adults with Fontan palliation and liver cirrhosis undergoing esophagogastroduodenoscopy (EGD).

**Method:**

The results of EGD, abdominal ultrasound, and liver biopsy, as well as clinic notes from the hepatologist, were reviewed to determine the diagnosis of cirrhosis and esophageal varices. The incidence of acute gastrointestinal bleeding complication was assessed among patients with esophageal varices using the time of EGD as the baseline.

**Results:**

Of 149 patients with Fontan palliation and liver cirrhosis, the prevalence of esophageal varices at baseline EGD was 34% (51 of 149). Of 98 patients without esophageal varices at baseline EGD, 27 (27%) underwent subsequent EGD, of whom 11 showed a new diagnosis of esophageal varices. The incidence of a new diagnosis of esophageal varices was 9% per year. Of 62 patients with esophageal varices, 9 (15%) had acute gastrointestinal bleeding complications during 45 (37-62) months of follow-up, yielding an incidence of 5% per year. Of the 9 patients, 8 underwent EGD and variceal banding during the hospitalization for bleeding and 1 patient died of septicaemia. Of the 8 patients who survived to hospital discharge, 2 patients were readmitted for esophageal bleeding within 12 months from the index hospitalization. Higher hepatic vein wedge pressure and hepatic vein pressure gradient were associated with esophageal varices and bleeding complications.

**Conclusions:**

In this selected sample of adults with Fontan palliation and liver cirrhosis, esophageal varices were relatively common, and patients with esophageal varices had risk of bleeding complications.

## Graphical abstract

**Figure:**
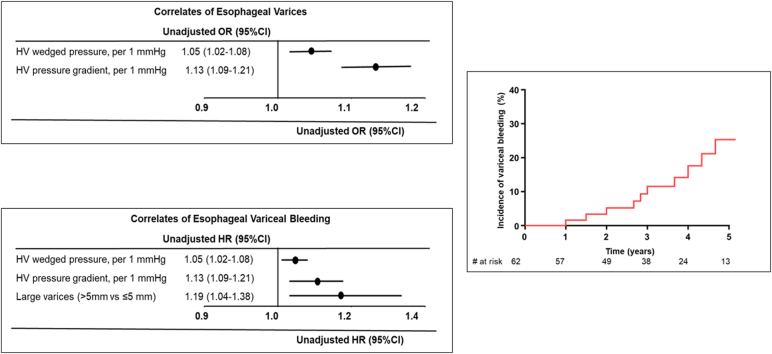

## Résumé

**Contexte:**

Cette étude visait à déterminer le risque de varices œsophagiennes et leur issue clinique chez les adultes ayant fait l’objet d’une intervention de Fontan et présentant une cirrhose hépatique chez qui une œsophagogastroduodénoscopie (OGD) a été réalisée.

**Méthodologie:**

Les résultats de l’OGD, de l’échographie abdominale et de la biopsie du foie, ainsi que les notes cliniques de l’hépatologue ont été consultés pour établir les diagnostics de cirrhose et de varices œsophagiennes. L’incidence des complications hémorragiques gastro-intestinales aiguës a été évaluée chez les patients présentant des varices œsophagiennes en utilisant l’OGD initiale comme référence de départ.

**Résultats:**

Chez les 149 patients ayant fait l’objet d’une intervention de Fontan et présentant une cirrhose hépatique, la prévalence des varices œsophagiennes lors de l’OGD initiale était de 34 % (51/149). Parmi les 98 patients sans varices œsophagiennes lors de l’OGD initiale, 27 (27 %) ont subi une OGD ultérieure, et 11 d’entre eux ont alors reçu un diagnostic de varices œsophagiennes. Le taux d’incidence des nouveaux diagnostics de varices œsophagiennes était de 9 % par année. Sur les 62 patients présentant des varices œsophagiennes, 9 (15 %) ont subi des complications hémorragiques gastro-intestinales aiguës au cours d’une période de suivi de 45 (37 à 62) mois, ce qui correspond à un taux d’incidence de 5 % par année. Huit des 9 patients ont subi une OGD et une ligature des varices par bande élastique durant leur hospitalisation en raison des complications hémorragiques, et un patient est décédé des suites d’une septicémie. Deux des 8 patients en vie au moment du congé de l’hôpital ont été réhospitalisés pour une hémorragie de l’œsophage dans les 12 mois suivant la première hospitalisation. Une pression d’occlusion plus élevée de la veine hépatique ainsi qu’un plus grand gradient de pression de la veine hépatique ont été associés à la survenue de varices œsophagiennes et de complications hémorragiques.

**Conclusions:**

Dans cet échantillon d’adultes ayant fait l’objet d’une intervention de Fontan et présentant une cirrhose hépatique, la fréquence des varices œsophagiennes était relativement élevée, et les patients présentant des varices œsophagiennes étaient exposés à un risque de complications hémorragiques.

Liver cirrhosis is common in adults with Fontan palliation because of chronic hepatic congestion inherent in the Fontan physiology.^1^, ^2^, ^3^, ^4^, ^5^, ^6^, ^7^ Chronic hepatic venous congestion leads to structural and functional remodelling, which in turn results in liver fibrosis and cirrhosis.^1^^,^^3^^,^^8^, ^9^, ^10^, ^11^ In health, the portal vein provides more than 70% of hepatic blood supply, and the portal circulation is unique because it relies on a low transhepatic gradient for hepatic blood flow (typically less than 5 mm Hg) to maintain liver perfusion and to decompress the splanchnic circulation.^12^, ^13^, ^14^ In contrast, the structural remodelling in liver cirrhosis results in an increased transhepatic gradient, and this in turn requires a higher portal venous pressure (portal hypertension) to generate the required pressure gradient to perfuse the liver.^12^, ^13^, ^14^ A chronic exposure to high portal venous pressure (portal hypertension) results in shunting of blood through the portosystemic circulation leading to formation of esophageal varices, which can sometimes present with life-threatening esophageal variceal bleeding.^12^, ^13^, ^14^

In spite of the high prevalence of cirrhosis among adults with Fontan palliation, there are limited data about the prevalence, risk factors, and outcomes of esophageal varices in this poplation.^5^^,^^15^ The purpose of this study was to define the clinical characteristics of patients with esophageal varices, identify correlates of esophageal varices, and delineate long-term clinical outcomes after the diagnosis of esophageal varices in adults with Fontan palliation undergoing esophagogastroduodenoscopy (EGD).

## Methods

### Study population

This is a retrospective cohort study of adults (aged ≥18 years) with Fontan palliation and liver cirrhosis who underwent EGD at the Mayo Clinic between January 1, 2003, and December 31, 2022. The diagnosis of liver cirrhosis was based on 2 criteria: abdominal ultrasound scan showing coarse echotexture consistent with chronic parenchymal disease and a clinical evaluation by the hepatologist confirming the diagnosis of cirrhosis.

All adults with Fontan palliation followed at the Mayo Clinic undergo an initial hepatology evaluation, typically at the time of initial presentation. This evaluation comprises a clinic visit with a Fontan hepatologist, abdominal imaging (ultrasound, computed tomography scan, or magnetic resonance imaging), and laboratory blood tests (comprehensive metabolic panel, complete blood count, hepatitis serology, and alpha-fetoprotein). Based on the outcome of this evaluation, some patients may be referred for EGD, invasive haemodynamic assessment, or liver biopsy. The patients with cirrhosis undergo abdominal ultrasound, comprehensive metabolic panel, and alpha-fetoprotein every 6 months and a clinical evaluation in a hepatology clinic every 1-2 years. The patients without cirrhosis undergo abdominal ultrasound, comprehensive metabolic panel, and alpha-fetoprotein every 12 months and a clinical evaluation in a hepatology clinic every 3-5 years.

The electronic health records were reviewed including clinic notes, procedure notes, echocardiogram, cardiac catheterization report, and laboratory data. The date of EGD was regarded as the baseline encounter, and the clinical data obtained within 6 months from the baseline encounter were used to define the baseline clinical characteristics.

### Study objectives

The aims of the study were to determine (1) the prevalence of esophageal varices in Fontan patients with liver cirrhosis undergoing EGD, and the correlates of esophageal varices; (2) the incidence of a new diagnosis of esophageal varices in subsequent EGDs in patients without esophageal varices identified in prior EGD; and (3) the incidence and risk factors for acute gastrointestinal (GI) bleeding complication during follow-up. Acute upper GI bleeding complication was defined as hematemesis associated with at least 2 of the following features: decrease in haemoglobin by ≥2 g/dL, need for blood transfusion, or urgent medical evaluation in the emergency department.^16^, ^17^, ^18^

### Data abstraction

The EGD reports and the clinic note from the hepatologist were reviewed to determine the presence, size, and location of esophageal varices. Based on these reports, esophageal varices were defined as large (>5 mm) or small (≤5 mm). Abdominal ultrasound reports were reviewed to determine the splenic size, presence of ascites, and presence of liver cirrhosis. Results of viral hepatitis serology and alcohol intake were retrieved from hepatology evaluation. Alcohol intake was classified as none (<1 drink/wk), light/moderate (1-14 drinks/wk), and heavy intake (>14 drinks/wk).^19^

Liver histologic data were abstracted from the pathology reports in the patients who underwent liver biopsy. The liver specimens were stained with trichrome and reticulin stains. Portal fibrosis was assessed using the Batts-Ludwig (stages 0-4) staging system, and sinusoidal fibrosis was staged (0-4) as previously described.^6^^,^^9^^,^^20^ Similar to previous studies,^6^ we dichotomized the patients into those with no sinusoidal fibrosis (stage 0) vs sinusoidal fibrosis (stages 1-4) and into patients without portal fibrosis (F0) vs with portal fibrosis (F1-4).

Cardiac catheterization was performed on chronic medications in the fasting state and under mild sedation.^21^^,^^22^ As previously described, venous access was obtained via internal jugular vein or femoral vein, and venous catheterization was performed using a 7 F balloon wedge catheter.^21^^,^^22^ Arterial catheterization was performed via a radial arterial or femoral arterial access using a 4 F or 5 F pigtail catheter for a direct measurement of arterial pressure and arterial oxygen saturation. Venous oxygen saturation was directly measured from samples drawn from the branch pulmonary arteries (mixed venous saturation) and pulmonary artery wedge position (pulmonary venous saturation). A saturation of 95% was used as the assumed pulmonary venous saturation in cases where the pulmonary venous saturation was not measured directly. Arterial saturation was measured from radial artery, femoral artery, or ventricular blood samples. Cardiac output was determined by the indirect Fick technique using assumed oxygen consumption and directly measured oxygen contents in the pulmonary artery and systemic circulations.^21^^,^^22^ For the assessment of hepatic haemodynamics, the catheter position in the hepatic vein was confirmed by appearance on fluoroscopy and contrast angiography before the measurement of free hepatic vein pressure and hepatic vein wedge pressure. Hepatic vein pressure gradient was calculated as hepatic vein wedge pressure minus free hepatic vein pressure.^3^

### Statistical analysis

Data were presented as median (interquartile range [IQR]), and count (%). Between-group comparisons were performed using the unpaired *t* test, Wilcoxon rank-sum test for continuous variables, and Fisher exact test. Logistic regression analysis was used to assess the correlates of esophageal varices. The cumulative incidence of acute GI bleeding complication was assessed using the Kaplan-Meier method and Cox regression. Missing data were addressed using conditional amputation.^23^ All statistical analyses were performed with the BlueSky Statistics software (version 7.10; BlueSky Statistics LLC, Chicago, IL), and a *P* value of <0.05 was considered to be statistically significant for all analyses.

## Results

### Baseline characteristics

Of 455 adult Fontan patients, 149 (33%) were considered to have the diagnosis of liver cirrhosis based on hepatology evaluation and were referred for EGD. The median age at the time of EGD was 32 (IQR: 25-39) years, and 85 (57%) were male. The initial Fontan connection was atriopulmonary Fontan connection in 76 (51%) patients, lateral tunnel/intra-atrial conduit Fontan in 40 (27%) patients, and extracardiac conduit Fontan in 33 (22%) patients. Of the 76 patients with atriopulmonary Fontan operation, 49 (64%) subsequently underwent Fontan conversion operation.

Of the 149 patients, 51 (34%) had esophageal varices at the time of baseline EGD. All 51 varices were located in the distal esophagus, and 14 (27%) patients had large esophageal varices (>5 mm). Of the 51 patients, 34 (67%) had associated portal gastropathy. Compared with the patients without esophageal varices, those with esophageal varices had a higher hepatic vein wedge pressure 18 (IQR: 15-20) mm Hg vs 15 (IQR: 13-17) mm Hg (*P* = 0.01) and a higher hepatic vein pressure gradient 3 (IQR: 2-4) mm Hg vs 1 (IQR: 0-2) mm Hg (*P* = 0.005) (Table 1). There were no other significant between-group differences in the clinical and haemodynamic characteristics of patients with vs without esophageal varices (Table 1).

**Table 1:** Baseline characteristics

Characteristic	All (N = 149)	Varices (N = 51)	No varices (N = 98)	*P*
Age, median (IQR) (y)	32 (25-39)	31 (24-37)	34 (26-39)	0.09
Male sex, n (%)	85 (57)	31 (61)	54 (55)	0.005
Surgical history				
Type of Fontan connection, n (%)				0.5
Atriopulmonary Fontan	76 (51)	24 (47)	52 (53)	
Lateral tunnel/IAC Fontan	40 (27)	15 (29)	25 (26)	
Extracardiac conduit Fontan	33 (22)	11 (22)	22 (23)	
Age at Fontan operation, median (IQR) (y)	6 (4-9)	5 (3-8)	6 (4-9)	0.3
Subsequent Fontan conversion, n (%)	49 (33)	16 (31)	33 (34)	0.4
Age at Fontan conversion, median (IQR) (y)	23 (17-29)	22 (17-28)	23 (19-29)	0.3
CHD diagnosis, n (%)				0.6
Tricuspid atresia	79 (53)	53 (54)	26 (51)	
Double inlet left ventricle	24 (16)	7 (14)	17 (17)	
Double outlet right ventricle	19 (13)	7 (14)	12 (12)	
Pulmonary atresia	11 (7)	5 (8)	6 (6)	
Hypoplastic left heart syndrome	6 (4)	2 (4)	4 (4)	
Others	10 (7)	4 (8)	6 (6)	
Other anatomic data, n (%)				
Systemic left ventricle	114 (77)	38 (75)	76 (78)	0.4
Heterotaxy	14 (9)	5 (10)	9 (9)	0.8
Comorbidities, n (%)				
Atrial arrhythmias	77 (52)	24 (47)	53 (54)	0.4
Protein losing enteropathy	21 (14)	5 (10)	16 (16)	0.3
Alcohol intake				0.7
None (0 drinks/wk)	88 (59)	31 (61)	57 (58)	
Light/moderate (1-14 drinks/wk)	61 (41)	20 (39)	41 (42)	
Heavy (>14 drinks/wk)	0	0	0	
Medications, n (%)				
Loop diuretics	77 (52)	24 (47)	53 (54)	0.4
β-Blockers	55 (37)	16 (31)	39 (40)	0.3
ACEI/ARB	76 (51)	27 (53)	49 (50)	0.7
MRA	55 (37)	19 (37)	36 (37)	0.9
Antiplatelet therapy	56 (38)	20 (39)	36 (37)	0.4
Anticoagulation	70 (47)	21 (41)	49 (50)	0.3
Vital signs				
Systolic blood pressure, mean ± SD (mm Hg)	109 ± 13	106 ± 11	112 ± 10	0.4
Diastolic blood pressure, mean ± SD (mm Hg)	68 ± 9	65 ± 7	70 ± 9	0.3
Heart rate, mean ± SD (mm Hg)	75 ± 13	78 ± 11	73 ± 10	0.5
Systemic oxygen saturation, median (IQR) (%)	92 (88-94)	91 (87-93)	93 (88-94)	0.3
NYHA III/IV, n (%)	55 (37)	14 (28)	41 (41)	0.2
Echocardiography	(N = 149)	(N = 51)	(N = 98)	
≥ Mod systemic ventricular dilation, n (%)	41 (28)	15 (29)	26 (28)	0.8
≥Estimated ventricular EF, median (IQR)	53 (48-58)	51 (46-56)	55 (49-58)	0.6
≥Mod systemic AVV regurgitation, n (%)	21 (14)	6 (12)	15 (15)	0.7
Cardiac catheterization, median (IQR)	(N = 141)	(N = 48)	(N = 93)	
Mixed venous saturation (%)	69 (62-74)	70 (63-74)	68 (61-73)	0.9
Systemic saturation (%)	91 (88-94)	92 (87-94)	91 (88-94)	0.5
Free HV pressure (mm Hg)	15 (13-18)	15 (13-18)	15 (12-17)	0.4
Wedge HV pressure (mm Hg)	16 (14-18)	18 (15-20)	15 (13-17)	0.01
HV pressure gradient (mm Hg)	2 (0-3)	3 (2-4)	1 (0-2)	0.005
Fontan pressure (mm Hg)	15 (13-18)	15 (13-19)	15 (12-17)	0.1
PAWP (mm Hg)	11 (8-14)	11 (8-14)	10 (9-15)	0.5
Transpulmonary gradient (mm Hg)	4 (2-5)	4 (2-5)	5 (2-6)	0.3
PVR index (WU m^2^)	1.91 (1.41-2.69)	1.86 (1.41-2.48)	1.94 (1.53-2.69)	0.2
Qs index (L/min/m^2^)	2.53 (2.10-2.98)	2.42 (2.10-2.74)	2.64 (2.25-3.03)	0.2

Between-group comparisons were based on the unpaired *t* test and Wilcoxon rank-sum test for continuous variables and the Fisher exact test for categorical variables.

ACEI/ARB, angiotensin-converting enzyme inhibitor/aldosterone receptor antagonist; AVV, atrioventricular valve; CHD, congenital heart disease; EF, ejection fraction; HV, hepatic vein; IAC, intra-atrial conduit; IQR, interquartile range; MRA, mineralocorticoid receptor antagonist; NYHA, New York Heart Association; PAWP, pulmonary artery wedge pressure; PVR, pulmonary vascular resistance; Qs, systemic blood flow; WU, Woods unit.

**Figure 1:**
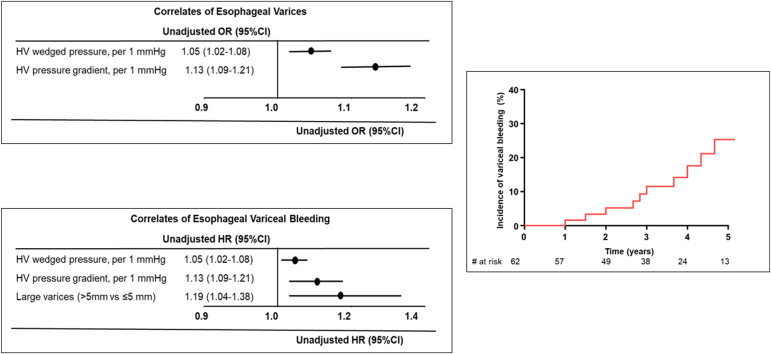
Forest plot showing correlates of esophageal varices based on univariable logistic regression analysis (**top left**). The forest plot showing correlates of esophageal variceal bleeding based on univariable Cox regression analysis (**bottom left**). The Kaplan-Meier curve showing the cumulative incidence of esophageal variceal bleeding (**right**). The variceal size was modelled as a binary variable, and large varices were defined >5 mm in size. CI, confidence interval; HR, hazard ratio; HV, hepatic vein; OR, odds ratio.

Table 2 shows comparisons of hepatic indices between the 2 groups. There were no significant between-group differences in liver function indices, abdominal ultrasound findings, magnetic resonance imaging–derived liver stiffness, and liver fibrosis on histology. On univariable analysis, hepatic vein wedge pressure (odds ratio: 1.05, 95% confidence interval [CI]: 1.02-1.08 per 1 mm Hg increase, *P* = 0.009) and hepatic vein pressure gradient (odds ratio: 1.13, 95% CI: 1.09-1.21 per 1 mm Hg increase, *P* = 0.006) were associated with the presence of esophageal varices on EGD (Table 3, Fig. 1).

**Table 2:** Hepatic indices

	All (N = 149)	Varices (N = 51)	No varices (N = 98)	*P*
Laboratory data, median (IQR)				
Haemoglobin (g/dL)	14.4 (12.8-15.6)	14.5 (12.7-15.6)	14.3 (12.5-15.5)	0.9
Platelet count (×10^9^/L)	153 (111-205)	129 (196-208)	166 (116-204)	0.9
Aspartate aminotransferase (U/L)	28 (23-39)	29 (24-40)	28 (23-39)	0.5
Alanine aminotransferase (U/L)	28 (22-37)	29 (22-36)	27 (22-37)	0.5
Alkaline phosphatase (U/L)	83 (68-114)	88 (76-122)	80 (65-112)	0.5
Albumin (g/dL)	4.4 (3.8-4.7)	4.3 (3.2-4.8)	4.4 (3.8-4.7)	0.5
Total bilirubin (mg/dL)	0.9 (0.6-1.4)	1.1 (0.7-1.5)	0.8 (0.6-1.3)	0.03
Direct bilirubin (mg/dL)	0.3 (0.2-0.4)	0.3 (0.2-0.5)	0.3 (0.2-0.4)	0.2
International normalized ratio	1.4 (1.2-2.1)	1.5 (1.2-2.1)	1.4 (1.2-2.0)	0.7
Creatinine (mg/dL)	0.9 (0.8-1.1)	1.0 (0.8-1.1)	0.9 (0.7-1.1)	0.2
Estimated GFR (mL/min/1.73 m^2^)	87 (73-109)	82 (69-102)	92 (76-112)	0.1
Alpha fetoprotein (ng/mL)	3 (1.5-4.2)	2.6 (1.5-3.7)	3 (1.7-4.4)	0.3
Hepatitis B/C, n (%)	7 (5)	2 (4)	5 (5)	0.6
Abdominal ultrasound	(N = 149)	(N =51)	(N = 98)	
Liver fibrosis/coarse echotexture, n (%)	149 (100)	51 (100)	98 (100)	0.9
Ascites, n (%)	43 (29)	17 (33)	26 (31)	0.5
Splenomegaly, n (%)	27 (53)	54 (55)	84 (56)	0.4
Splenic size, median (IQR) (mm)	14.7 (13.1-16.4)	14.6 (13.2-17.2)	14.7 (13.1-16.0)	0.5
Magnetic resonance elastography	(N = 86)	(N = 36)	(N = 50)	
Liver stiffness, median (IQR) (kPa)	4.9 (4.1-5.5)	5.2 (4.3-5.4)	4.6 (3.8-5.6)	0.3
Liver biopsy, n (%)	(N = 56)	(N = 19)	(N = 37)	
Sinusoidal fibrosis (categories 1-4)	55 (98)	19 (100)	36 (97)	0.7
Portal fibrosis (categories 1-4)	52 (93)	18 (95)	34 (92)	0.6

Between-group comparisons were based on the unpaired *t* test and Wilcoxon rank-sum test for continuous variables and the Fisher exact test for categorical variables.

GFR, glomerular filtration rate.

**Table 3:** Univariable logistic regression models for correlates of esophageal varices

	OR (95% CI)	*P*
Demographic/anatomic indices		
Age (y)	0.97 (0.93-1.01)	0.1
Male sex	1.75 (0.82-3.72)	0.1
Atriopulmonary Fontan	0.98 (0.88-1.06)	0.3
Systemic left ventricle	1.01 (0.74-1.26)	0.5
Others		
Hepatitis B/C	0.98 (0.87-1.14)	0.6
Alcohol intake (light/moderate vs none)	1.02 (0.93-1.12)	0.4
Medications		
β-Blocker therapy		
None	Reference	–
Selective β-blocker therapy	0.82 (0.65-1.09)	0.2
Nonselective β-blocker therapy	0.98 (0.84-1.11)	0.6
Echocardiographic indices		
Estimated ventricular ejection fraction	0.98 (0.93-1.02)	0.3
≥Moderate AVV regurgitation	0.74 (0.18-2.93)	0.7
Abdominal ultrasound		
Ascites	1.03 (0.46-3.15)	0.6
Splenic size (mm)	1.07 (0.91-1.26)	0.4
Cardiac catheterization		
Wedge HV pressure (mm Hg)	1.05 (1.02-1.08)	0.009
HV pressure gradient (mm Hg)	1.13 (1.09-1.21)	0.006
Fontan pressure (mm Hg)	1.11 (0.99-1.25)	0.07
PAWP (mm Hg)	1.05 (0.96-1.14)	0.3
PVR index (WU m^2^)	1.02 (0.98-1.04)	0.4
Qs index (L/min/m^2^)	1.46 (0.90-2.39)	0.1

β-Blocker therapy was modeled as a categorical variable using the patients who were not on any therapy as the reference group.

AVV, atrioventricular valve; CI, confidence interval; HV, hepatic vein; OR, odds ratio; PAWP, pulmonary artery wedge pressure; PVR, pulmonary vascular resistance; Qs, systemic blood flow.

At the time of the diagnosis of esophageal varices, 16 (31%) patients were on β-blocker therapy (selective β-blocker n = 14 and nonselective n = 2). Nonselective β-blocker therapy was initiated in another 29 (57%) patients. Of the other 6 patients, 4 patients had a prior history of intolerance to an initial trial of β-blocker therapy for atrial arrhythmias, mostly because of chronotropic incompetence and severe fatigue. The remaining 2 patients have no prior history of β-blocker use as can be determined from the review of medical records. There was no statistically significant difference in the proportion of patients on β-blocker therapy at the time of EGD among the patients with esophageal varices vs patients without esophageal varices (16 of 51 [31%] vs 39 of 98 [40%], *P* = 0.3).

### New diagnosis of esophageal varices on subsequent EGD

Of 98 patients without esophageal varices on baseline EGD, 27 (27%) underwent subsequent EGD, and the median interval between the first and the second EGD was 51 (IQR: 43-86) months. The EGDs were performed as part of the routine Fontan monitoring protocol, and none of the patients had a prior history of GI bleeding. Of the 27 patients, 11 (41%) had a new diagnosis of esophageal varices on the second EGD. All 11 patients had small esophageal varices involving the lower third of the esophagus, and 6 patients had associated portal gastropathy. The incidence of a new diagnosis of esophageal varices was 9% per year. At the time of the diagnosis of esophageal varices, 4 of the 11 (36%) patients were on selective β-blocker therapy, and nonselective β-blocker therapy was initiated in another 7 patients.

### Acute GI bleeding complications and interventions

Of the 62 patients with esophageal varices, 9 (15%) had acute upper GI bleeding complications during a median follow-up of 45 (IQR: 37-62) months, yielding an incidence of 5% per year. The 5-year cumulative incidence of esophageal variceal bleeding among patients with esophageal varices was 26% (95% CI: 19-33) (Fig. 1). On univariable analysis, the correlates of acute GI bleeding complications were the presence of large esophageal varices on EGD (hazard ratio [HR]: 1.19, 95% CI: 1.04-1.38, *P* = 0.02) and hepatic vein wedge pressure (HR: 1.05, 95% CI: 1.05-1.08, *P* = 0.01), and hepatic vein pressure gradient (HR: 1.13, 95% CI: 1.09-1.21, *P* = 0.007) (Table 4, Fig. 1). Of note, the β-blocker therapy was not associated with the GI bleeding complications.

**Table 4:** Univariable Cox regression models for correlates of upper GI bleeding

	HR (95% CI)	*P*
Demographic/anatomic indices		
Age (y)	1.08 (0.92-1.34)	0.4
Male sex	1.33 (0.862.92)	0.4
Atriopulmonary Fontan	1.14 (0.83-178)	0.3
Systemic left ventricle	1.04 (0.79-133)	0.4
Others		
Hepatitis B/C	0.98 (0.87-1.14)	0.6
Alcohol intake (light/moderate vs none)	1.02 (0.93-1.12)	0.4
Medications		
β-Blocker therapy		
None	Reference	–
Selective β-blocker therapy	0.98 (0.87-1.13)	0.3
Nonselective β-blocker therapy	0.93 (0.78-1.09)	0.1
Echocardiographic indices		
Estimated ventricular ejection fraction	0.88 (0.98-1.09)	0.4
≥Moderate AVV regurgitation	1.82 (0.64-3.04)	0.7
Abdominal ultrasound		
Ascites	1.14 (0.88-3.4)	03
Splenic size (mm)	1.04 (0.94-1.19)	0.5
EGD findings		
Large esophageal varices	1.19 (1.04-1.38)	0.02
Portal gastropathy	1.05 (0.92-1.14)	0.3
Cardiac catheterization		
Wedge HV pressure (mm Hg)	1.05 (1.02-1.08)	0.01
HV pressure gradient (mm Hg)	1.13 (1.09-1.21)	0.007
Fontan pressure (mm Hg)	1.06 (0.98-1.10)	0.08
PAWP (mm Hg)	1.07 (0.92-1.17)	0.2
PVR index (WU m^2^)	1.23 (0.87-1.51)	0.5
Qs index (L/min/m^2^)	1.28 (0.94-1.86)	0.4

β-Blocker therapy was modeled as a categorical variable using the patients who were not on any therapy as the reference group.

AVV, atrioventricular valve; CI, confidence interval; EGD, esophagogastroduodenoscopy; HR, hazard ratio; HV, hepatic vein; PAWP, pulmonary artery wedge pressure; PVR, pulmonary vascular resistance; Qs, systemic blood flow.

All 9 patients with GI bleeding complications were hospitalized. The median decline in haemoglobin concentration was 2.4 (IQR: 1.7-3.1) g/dL, and 8 of the 9 patients required blood transfusion. Of the 9 patients, 8 underwent EGD and variceal banding during the index hospitalization (hospitalization for acute GI bleeding). One of the 9 patients with variceal bleeding died during the index hospitalization for esophageal bleeding because of septicaemia and hepatic encephalopathy. Of the 8 patients who survived to hospital discharge, 2 patients were readmitted for GI bleeding within 12 months from the index hospitalization.

## Discussion

In this study, we assessed the prevalence, correlates, and outcomes of esophageal varices among adults with Fontan palliation and liver cirrhosis who underwent EGD. The main findings were as follows: (1) The prevalence of esophageal varices at baseline EGD was 34%. (2) Among patients without esophageal varices at baseline EGD, the annual incidence of new diagnosis esophageal varices on subsequent EGDs was 9% per year. (3) The correlates of esophageal varices diagnosis (at baseline and subsequent EGD) were higher hepatic vein wedge pressure and higher hepatic vein pressure gradient. (4) Among patients with the diagnosis of esophageal varices, the annual incidence of acute GI bleeding complication was 5% per year, and the 5-year cumulative incidence of major upper GI bleeding complication was 26%. (5) The correlates of acute GI bleeding complications were large esophageal varices, higher hepatic vein wedge pressure, and higher hepatic vein pressure gradient.

Chronic liver disease is relatively common in adults with Fontan palliation, and the prevalence of liver cirrhosis has been estimated at 20%-30% in this population.^1^, ^2^, ^3^, ^4^, ^5^, ^6^, ^7^ Despite the high prevalence of cirrhosis among adults with Fontan palliation, there are limited data about the prevalence, risk factors, and outcomes of esophageal varices in this population.^1^, ^2^, ^3^, ^4^, ^5^^,^^9^^,^^24^ The available data suggest that the presence of esophageal varices, in combination with other features such as ascites, splenomegaly, and thrombocytopenia, was associated with adverse outcomes.^5^ To the best of our knowledge, this is the first systematic analysis of the disease burden of esophageal varices among adults with Fontan palliation and liver cirrhosis. Although the analysis was based on a selected population, it provides clinically relevant estimates of disease prevalence and bleeding risk of esophageal varices in this population.

These findings have important clinical implications with regards to monitoring of adults with Fontan physiology. Currently, the only definitive treatment for Fontan-associated liver disease is heart-liver transplant.^25^^,^^26^ However, this therapy is often reserved for patients with advanced or end-stage disease.^25^^,^^26^ For the majority of patients with Fontan physiology who do not meet criteria for heart-liver transplant, medical management centres around the treatment of reversible haemodynamic lesions (surgical or transcatheter therapy), treatment of complications of portal hypertension using medical therapy (diuretics and β-blockers), and paracentesis, and close surveillance for liver-related complications such as hepatocellular carcinoma and esophageal varices.^4^^,^^27^ The results of the current study demonstrating esophageal varices in one-third of adult Fontan patients with liver cirrhosis suggest that a baseline EGD should be performed in all adults Fontan patients with cirrhosis. Fontan patients without liver cirrhosis were excluded from the current study, and hence we are unable to comment on the diagnostic yield or clinical benefit of screening for esophageal varices in that subset of patients.

Another important clinical implication of the results of this study is the relatively high incidence of esophageal variceal bleeding in this population. In the non-Fontan population, the detection of esophageal varices provides opportunity for prophylactic therapy such as use of nonselective β-blocker therapy, somatostatin analog, and esophageal variceal banding.^12^^,^^28^^,^^29^ These therapies have been shown to decrease portal venous pressure and reduce the risk of acute GI bleeding based on data from the non-Fontan population.^12^^,^^28^^,^^29^ In the current study, we did not observe any association between β-blocker therapy and esophageal varices, but these results should be interpreted with caution because the study was not designed to test the efficacy (or lack thereof) of these prophylactic therapies.

### Limitations

This is a retrospective, single-centre cohort study, and it is therefore prone to selection and ascertainment bias. The use of β-blocker therapy or the criteria for esophageal variceal banding were not standardized. Finally, we did not adjust for potential confounders in the statistical model because of the small sample size. These limitations may limit the generalizability and reproducibility of the results.

## Conclusions

Esophageal varices were relatively common in adults with Fontan palliation and liver cirrhosis and were associated with the risk of acute GI bleeding complications. These data support the need for screening for esophageal varices among patients with Fontan palliation and liver cirrhosis. Further studies are required to assess opportunities to prevent the development of varices, the diagnostic yield of less invasive screening strategies such as abdominal computerized tomography scan, and to determine the optimal therapy to prevent life-threatening bleeding in patients with esophageal varices. It is important to emphasize that these data were derived from a selected patient population with liver cirrhosis, and hence the prevalence of esophageal varices (and in turn the yield of EGD) maybe lower in other cohorts.
